# Identification of Cacao Mild Mosaic Virus (CaMMV) and Cacao Yellow Vein-Banding Virus (CYVBV) in Cocoa (*Theobroma cacao*) Germplasm

**DOI:** 10.3390/v13112152

**Published:** 2021-10-26

**Authors:** Ihsan Ullah, Andrew J. Daymond, Paul Hadley, Michelle J. End, Pathmanathan Umaharan, Jim M. Dunwell

**Affiliations:** 1 School of Agriculture, Policy and Development, University of Reading, Reading RG6 6EU, UK; i.ullah@reading.ac.uk (I.U.); a.j.daymond@reading.ac.uk (A.J.D.); p.hadley@reading.ac.uk (P.H.); 2 Cocoa Research Association; michelle.end@cocoareserch.org.uk; 3 Cocoa Research Centre, St. Augustine Campus, The University of the West Indies, Trinidad and Tobago; Pathmanathan.Umaharan@sta.uwi.edu

**Keywords:** badnavirus, cocoa, LAMP assay

## Abstract

Cocoa, *Theobroma cacao*, is an important tropical perennial crop grown widely in the humid tropics. The exchange of cocoa germplasm between germplasm collections and breeding centres is vital for varietal development. Intermediate quarantine facilities, such as the International Cocoa Quarantine Centre, Reading UK (ICQC-R) play a vital role in ensuring the transfer of germplasm whilst minimising the risk of spreading pests and diseases. Current screening procedures combine visual inspection and molecular techniques, which are effective in detecting Cocoa swollen shoot virus (CSSV), a badnavirus, which causes severe losses but are restricted to West Africa. However, the detection of latent or mild virus infections that produce no visual symptoms has been a challenge. Recently two badnavirus species of cocoa producing mild symptoms, cacao mild mosaic virus (CaMMV) and cacao yellow vein-banding virus (CYVBV), have been sequenced. Here, we report new assays for the detection of these two species, for the first time in non-symptomatic accessions. Evolutionary and bioinformatic analyses of the viruses suggest their most recent source was from Trinidad, though there is historic evidence that these viruses may have their origin in South America and then become widespread globally over the last century. We also report a novel colorimetric Loop-mediated isothermal amplification (LAMP) assay for the detection of CYVBV. This simple and accurate method could be employed in field virus testing.

## 1. Introduction

Cocoa (*Theobroma cacao* L.), a perennial species originating in the Amazon basin, is globally known for being the raw material of chocolate and is grown by smallholder farmers mainly in humid tropical areas in West Africa, Central and South America, and South East Asia. Along with other biotic and abiotic factors, sustainable production is threatened by a range of fungal and virus diseases, many of which are endemic to certain cocoa producing regions but not others. The exchange of cocoa germplasm between genebanks and research institutes is vital for breeding improved planting material but carries the risk of concomitant spread of these pathogens. Viruses pose a particular challenge especially since some species can remain latent for long periods or cause very mild or no symptoms in their hosts. The International Cocoa Quarantine Centre at the University of Reading (ICQC-R) uses an internationally accepted virus-indexing procedure developed to detect Cocoa swollen shoot virus (CSSV), which causes severe crop losses and tree death in many cocoa producing areas of West Africa [1]. The virus indexing procedure is based on grafting the test material onto indicator rootstocks of the Amelonado variety and visual observation for symptom development over a two-year period. In recent years, the quarantine procedure has been strengthened by the introduction of PCR testing using primers developed to detect the known CSSV species whilst taking into account the integrated viral sequences known to occur in a wide range of cocoa germplasm [2]. 

The quarantine procedures in place at ICQC-R have successfully intercepted suspected viruses on a number of occasions. For example, virus indexing in 2004 of a clone received from Trinidad in 2002 led to the subsequent molecular characterization of two badnaviruses in samples from the International Cocoa Genebank, Trinidad (ICG-T), named cacao mild mosaic virus (CaMMV) and cacao yellow vein-banding virus (CYVBV) [3]. These viruses correspond to two distinctive virus-like symptoms detected in Trinidad in the 1940s and putatively designated as caused by Cocoa Trinidad Virus (CTV) Strain A and Strain B, based on their symptomology; CTV-Strain A (now CaMMV) causing red foliar mottling and CTV-Strain B (now CYVBV) causing yellow vein-banding on cocoa leaves. Research on the virus in Trinidad during the 1940–1950s showed that it could be transmitted by grafting or via a mealybug vector, and that there were some genotypic differences in symptom expression [4,5]. However, no further studies were undertaken since it appeared that the government mandated-eradication program in the 1950s had successfully contained the virus outbreak to a limited number of cocoa-growing areas on the island and subsequently eliminated it through cutting out of infected trees. No reports of CTV were made on either Trinidad or Tobago until virus-like symptoms were observed on a limited number of trees held in the ICG-T in 2003. 

Although the molecular characterization of CaMMV and CYVBV provided the first identification of cocoa-infecting badnaviruses in the Western Hemisphere, with recent reports confirming the presence of CaMMV in commercially grown trees in Brazil and Puerto Rico [6], there are several earlier reports of virus-like symptoms in other cocoa growing areas in the region, including Colombia and Dominican Republic [7] and Venezuela [8], and it has been hypothesized that CaMMV and its close relatives may be more widespread than previously thought, both in cocoa germplasm and in cocoa cultivated throughout the tropical Americas. 

There have also been sporadic reports of viral symptoms on cocoa plants in South East Asia over the last 50 years or so, though there has been little research undertaken due to the low impact of the disease. For example, Semangun [9] reported that, although viral symptoms were widespread in cocoa growing in Java, suggesting that the disease had been present for a long time, it had attracted little attention since it did not cause heavy crop losses. Kenten and Woods (1976) [10] examined material of a Trinitario clone from North Sumatra and described the isolation of bacilliform virus particles transmissible to Amelonado plants, by use of a mealybug vector. The sampled material seemed to tolerate the virus well and only occasionally showed symptoms which ranged from clearing of the major veins to a more complex mosaic pattern; no stem swellings were observed. They also conclude “It is of interest that Legg [11] reported the presence of a mealybug transmitted virus in clones DR1 and DR38 received in Ghana after many years of intermediate quarantine at Wageningen, Holland, during which no virus symptoms were seen. It seems likely that these clones imported into Holland from Indonesia were infected with the same virus as that described here”.

Subsequently, Turner and Shepherd [12], in a summary of cocoa diseases in Indonesia and Malaysia, conclude that “In Indonesia, virtually total use in the past of Trinitario material may have restricted the occurrence of certain diseases, although it seems certain that a widespread virus disease has long been present”. They also comment that “Within the Trinitario population the appearance of symptoms is extremely variable, sometimes being readily visible in many leaves at one time but barely observable during the following year”. Additionally, they suggest that the widespread occurrence of a virus of the swollen shoot group in Indonesia has created uncertainty because of what might happen if it is transferred from its apparently tolerant Trinitario host to the newly introduced Upper Amazon hybrids. 

Lockwood [13] described the discovery in 1991 of a “fern leaf” symptom on the introduced clone GS 50 growing at four locations in Sabah. He stated that this clone, which is of Caribbean origin, had passed intermediate quarantine in the USDA Sub-Tropical Horticultural Research Station (SHRS) Miami, Florida and post-entry quarantine at the Highlands Research Unit (HRU) in Peninsular Malaysia. In addition, he compared this finding with the observation in Ghana in 1971–1975 of a “fern leaf” symptom on clone DR 38 from Indonesia. This symptom had been shown to be associated with a mealybug transmissible agent. In a situation similar to that of clone GS 50, the clone DR 38 had passed through intermediate quarantine in greenhouse facilities in Wageningen, The Netherlands. In a prescient comment that is still pertinent today, 30 years later, Lockwood (1992) [13] concludes that “This highlights the need for a concentrated effort on the development of rapid, and reliable (if possible) diagnostic techniques. There is need for dialogue between the quarantine stations and receiving countries as to what procedures are both practicable and reasonably effective”. 

The most recent report from this region, and one related directly to the finding in the present study, is that concerning clone BR 25 (ICQC-R Accession RUQ 1739) [14]. Material from this clone was sent to ICQC-R from the Malaysian Cocoa Board in 2017 and grafted on to Amelonado test plants as part of the virus-indexing process. Less than two months later, some of these test plants showed virus symptoms, whilst the plants were still in isolation and all the material was consequently destroyed.

It is important to note that the accurate diagnosis of any virus, and specifically those that produce few if any symptoms in the host, is entirely dependent on the availability of their genome sequence. Therefore, although the currently recommended virus-indexing procedure at the ICQC-R has been successful in detecting the occurrence of mild strain viruses on a number of occasions, the recent availability of genomic data for the CaMMV and CYVBV species has provided an opportunity to screen the material for asymptomatic infections using standard PCR techniques. The genome sequences also allowed for the development of a novel Loop-mediated isothermal amplification (LAMP) assay. This type of assay, notable for its ease of use and high accuracy, is used routinely in diagnostic studies in animals and plants but has never previously been applied to cocoa.

In addition, following the precedent of our discovery of badnaviral sequences integrated into the cocoa genome [2] we also detected the presence of CYVMV and CaMMV sequences in a range of genomic datasets generated from a diverse range of global studies. Taken together, these molecular and bioinformatic analyses provide insight into the current distribution of these viruses both in the Americas and South East Asia. Additionally, we consider possible ways in which these viruses have spread around cocoa growing areas worldwide. 

## 2. Materials and Methods

### 2.1. PCR Screening of Cocoa Germplasm

#### 2.1.1. Plant Material

Asymptomatic and symptomatic leaves of cocoa accession ICS 27 (Tree ID 6B E286 T13) and ICS 76 (Tree ID 6B B109 T9), and a leaf of healthy cocoa accession RIM 52 [Tree ID 5A N(6) 636 T1] were obtained from ICG-T, managed by the Cocoa Research Centre of the University of the West Indies, Trinidad and Tobago, for optimization and validation of the PCR assays. For screening of germplasm in the ICQC-R, a fully expanded young leaf was sampled from each of the 352 accessions.

#### 2.1.2. DNA Extraction

Total genomic DNA was isolated from cocoa leaves using the DNeasy mini or DNeasy 96 Plant Kit (Qiagen, UK) following the manufacturer’s instructions. One hundred milligrams of leaf sample were ground with liquid nitrogen in a microcentrifuge tube in the presence of ceramic beads using TissueLyser II (Qiagen, Manchester, UK). The quality and quantity of isolated genomic DNA were determined using a NanoDrop 2000 Spectrophotometer (Thermo Fisher Scientific, Paisley, UK).

#### 2.1.3. Primer Design

Primer sets (Table S1) for standard PCR were designed on the RT-RNase H region of CYVBV (Accession No. KX276641) and CaMMV (Accession No. KX276640) using Primer3Plus software (https://www.bioinformatics.nl/cgi-bin/primer3plus/primer3plus.cgi). 

#### 2.1.4. PCR Conditions

For PCR, a 20 μL reaction was assembled containing 10 μL of Platinum™ hot start PCR master mix (Thermo Fisher Scientific, UK), 2 μL each of 5 μM primer and 6 μL of 10ng/μL DNA template. PCR reactions were performed in a thermal cycler (Veriti, Applied Biosystems, Paisley, UK) programmed to one cycle of 94 °C for 2 min, followed by 35 cycles of 94 °C for 15 s, and then either 64 or 62 °C for 15 s and 68 °C for 30 or 60 s for primer set one and set two, respectively. The PCR products were resolved on a 1.2 or 2% agarose gel and stained with ethidium bromide. GeneRuler 100 bp or 1 kb DNA ladder was used as a size standard. The PCR products were purified using GeneJET gel extraction and a DNA clean-up micro kit (Thermo Fisher Scientific, Paisley, UK) following the manufacturer’s instructions and were sequenced by Sanger technology (Source Bioscience, Cambrigde, UK). An accession was scored positive where we found a single discrete band of expected size verified by sequencing.

### 2.2. Development of LAMP Assay for Detection of CYVBV

#### 2.2.1. Primer Design

LAMP primer sets used in this study were designed from RT-RNase H region of CYVBV genome (Accession No. KX276641) using NEB LAMP primer design tool (https://lamp.neb.com/#!/). The primers were synthesized by Sigma-Aldrich, Dorset, UK. The sequences of primers are provided in Table S2.

#### 2.2.2. LAMP Assay

The LAMP reactions were assembled in 25 μL volume using 12.5 μL of WarmStart^®^ Colorimetric LAMP 2X master mix with UDG (M1804S, New England Biolabs, UK), 2.5 μL of 10 x primer mix including 16 μM FIP and BIP, 4 μM LF and LB, and 2 μM F3 and B3, and 50 ng of DNA or nuclease-free water for non-template controls (NTCs), in a total volume of 25 μL. Reactions were supplemented with nuclease-free water to bring the reaction volume to 25 μL. The reactions were incubated at 65 °C or as mentioned, in a thermal cycler (Veriti, Applied Biosystems, UK) for 30–45 min with the lid heated to 90 °C. Upon completion of the assay, the reaction tubes were transferred to an ice-cold metal block for 1 min, placed on a whiteboard and imaged using a smartphone with default setting. The LAMP reaction products were also analysed on a 2% agarose gel for confirmation of amplification.

### 2.3. Identification of CYVBV and CaMMV Viruses in Genomic Sequencing Datasets of Cocoa

Infection of cocoa accessions with CYVBV and CaMMV was first reported from samples collected in Trinidad and Tobago. The complete genomes of both viruses were also first identified and characterized within the Trinitario varietal group in the ICG-T. Recently, CaMMV genomes have been discovered in trees on commercial farms in Puerto Rico and Brazil. Hence, the Sequence Read Archive (SRA, https://www.ncbi.nlm.nih.gov/sra) was searched for the projects that report sequencing data for cocoa accessions from Brazil, Puerto Rico or Trinidad. Three studies that contain 240 raw reads datasets were selected (Table S3). The first BioProject selected was PRJNA486150 [15], which comprises raw reads datasets generated by reduced representation library sequencing from 30 *T. cacao* accessions from the collection at the ICG-T. The second selected BioProject, PRJNA77799 [16], contains ten Whole Genome Sequencing (WGS) datasets, nine *T. cacao* and one *T. grandiflorum*, mostly from the USDA Tropical Agriculture Research Station (TARS), Mayaguez, Puerto Rico. The third selected BioProject PRJNA486011 [17] contains 200 WGS datasets, including from accessions held at the ICG-T. 

For each BioProject, the raw read files, in FastQ format, were downloaded from the ENA (https://www.ebi.ac.uk/ena/browser/home, last accessed on 14 July 2021). The short reads were mapped using Bowtie2 v. 2.3.4.1 [18]. Nucleotide sequences of CYVBV isolate ICS27 (Accession No. KX276641) and CaMMV isolates SCA6, PR3, BR321 and BR322 (Accessions No. KX276640, MW052520-MW052522) genomes were retrieved from the NCBI (https://www.ncbi.nlm.nih.gov/nuccore/, last accessed on 14 July 2021) and used as the reference local database. The mapped reads were compressed, sorted and indexed by Samtools v. 1.101 [19]. The alignment data were visualised in the Integrative Genomics Viewer (IGV) v. 2.4.13 [20]. 

### 2.4. Sequence Assembly, Alignment and Evolutionary Analysis

The sequencing data were assembled using SeqMan Ultra contig assembly software (DNASTAR). The CLC sequence viewer (Qiagen, UK), Sequence Demarcation Tool version 1.2 [21] and MEGA7 [22,23] software were used for sequence alignment and evolutionary analysis.

## 3. Results

### 3.1. PCR Detection of CaMMV and CYVBV

**Figure 1:**
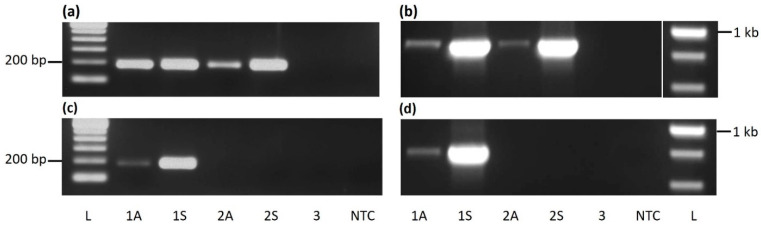
PCR-based detection of cacao mild mosaic virus (CaMMV) and cacao yellow vein-banding virus (CYVBV) in cocoa. A 193 bp (**a**) and a 861 bp (**b**) fragment of CaMMV, and a 183 bp (**c**) and a 802 bp (**d**) fragment of CYVBV targeting RT-RNase H region were amplified from cocoa accessions ICS 27 (1), ICS 76 (2) and RIM 52 (3) received from Trinidad. Letters A or S represents asymptomatic and symptomatic leaves, respectively. NTC stands for no template control and letter L denotes the lane loaded with 100 bp (**a**,**c**) or 1 Kb ladder (**b**,**d**).

Two primer sets were designed from the highly conserved RT-RNase H region to detect CaMMV DNA in cocoa accessions through PCR. The first set amplifies a 193 bp fragment (Figure 1a), whereas the second set targets an 861 bp region (Figure 1b). Both sets successfully amplified an expected size band from symptomatic as well as asymptomatic leaf samples from accessions ICS 27 and ICS 76. Relatively low amplification was found in the asymptomatic ICS 76 sample in PCR using the primer set 1. Amplification varied according to the amplicon size and virus titre. A negative correlation was found between amplification and fragment size. The variation in amplification between asymptomatic and symptomatic samples was more obvious for the large fragment size amplified with primer set 2.

Similarly, two primer sets were designed for the detection and identification of CYVBV; these targeted a 183 or 802 bp region of the RT-RNase H locus. A single amplicon of expected size was amplified in both assays from symptomatic as well as asymptomatic samples from ICS 27. No band was amplified from the ICS 76 sample in either assay. As expected, significantly lower amplification was found in the asymptomatic sample (Figure 1c,d). 

No amplification was found in the RIM 52 sample or no template control in any of the four assays.

### 3.2. Sequences of Amplified Fragments and Their Phylogenetic Relationship to Other Isolates

**Figure 2:**
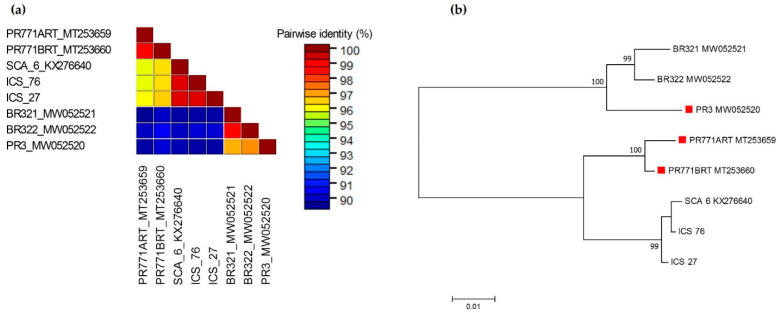
Molecular phylogenetic analyses of eight 861 bp nucleotide sequences of cacao mild mosaic virus (CaMMV) RT-RNase H region obtained in this study (ICS 27; ICS 76), extracted from CaMMV complete genome of isolates from Brazil (BR321 MW052521; BR322 MW052522), Puerto Rico (PR3 MW052520) and Trinidad (SCA 06 KX276640), and CaMMV polyprotein gene, partial cds of isolates from Puerto Rico (PR771ART MT253659; PR771BRT MT253660). (**a**) Pairwise comparisons were conducted using Sequence Demarcation Tool version 1.2 [21] (**b**) The evolutionary history was inferred by MEGA7 [22] by using the Maximum Likelihood method based on the Kimura 2-parameter model [23]. The percentage of trees in which the associated taxa clustered together is shown next to the branches. A discrete Gamma distribution was used to model evolutionary rate differences among sites. The tree is drawn to scale, with branch lengths measured in the number of substitutions per site. Red square before taxa names represent CaMMV isolates identified from RIM 52 accession in Puerto Rico.

In the case of CaMMV, no variation was found between the nucleotide sequence obtained from asymptomatic and symptomatic leaves of accessions ICS 27 and ICS 76. There was 99.5% identity between nucleotide sequences obtained from these two accessions and both sequences shared 99.4–99.5% nucleotide sequence identity with the CaMMV genome isolate reported previously from Trinidad [3] (GenBank accession KX276640). Comparison of the nucleotide sequences of ICS 27 and ICS 76 isolates with the recently reported CaMMV isolates in Brazil and Puerto Rico revealed interesting results. Three CaMMV sequences, namely, one complete genome isolate (GenBank accession MW052520) and two isolates of partial cds of the polyprotein gene (GenBank accessions MT253659; MT253660), identified from symptomatic trees of accession RIM 52, have been reported in Puerto Rico [6,24]. These isolates are designated as PR3, PR771ART and PR771BRT, respectively. Isolates from ICS 27 and ICS 76 shared 95.9 and 96.4% nucleotide identity with PR771ART and PR771BRT isolates, respectively, whereas they showed a relatively low nucleotide identity of 88.7–89.0% with the complete genome isolate PR3 (Figure 2a; supplementary Figure S1). Two CaMMV complete genome isolates (GenBank accessions MW052521, MW052522) have also been reported in Bahia state in Brazil, from symptomatic trees of accession VB 1151, designated as BR321 and BR322, respectively [6]. The ICS 27 and ICS 76 isolates had 88.8–89.2% and 89.0–89.5 nucleotide identity with the BR321 and BR322 isolates, respectively (Figure 2a). The phylogenetic analysis grouped the eight isolates in two major clades. One clade is formed by three genome isolates, two identified in Brazil and one identified in Puerto Rico. The second clade formed two distinct sister clades. The sequences reported in the present study, and the genome sequence reported in Trinidad, grouped in a basal sister clade, whereas the two isolates of partial cds of the polyprotein gene identified in Puerto Rico formed the second sister clade (Figure 2b).

In the case of CYVBV, no variation was found between nucleotide sequences obtained from asymptomatic and symptomatic leaves of accession ICS 27, except that three heterozygous SNPs were found in the sequence obtained from the asymptomatic sample. The nucleotide sequence of CaMMV in the isolate ICS 27 identified in this study was 99% identical with the genome of this virus (GenBank accession KX276640) identified in the same accession in Trinidad (Supplementary Figure S2).

### 3.3. Screening of ICQC-R Accessions

Visual inspection of the ICQC-R, germplasm collection failed to find any accession with CaMMV and CYVBV-like disease symptoms of the type described in the recent studies. Therefore, DNA isolated previously from a randomly selected fully expanded young leaf was used for PCR screening of germplasm using diagnostic primers for CaMMV and CYVBV. 

**Figure 3:**
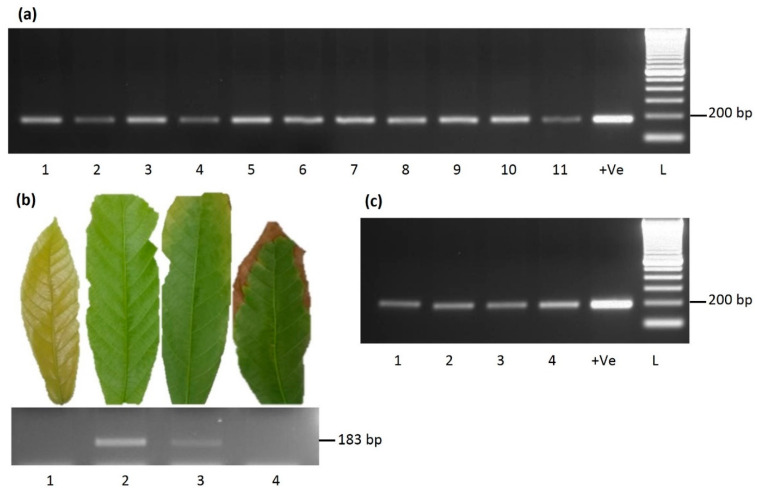
PCR based detection of cacao yellow vein-banding virus (CYVBV) and cacao mild mosaic virus (CaMMV) in cocoa. (**a**) A 183 bp fragment of RT-RNase H region of CYVBV amplified from cocoa accessions GU 144/C (1), GU 219/F (2), GU 265/P (3), GU 341/H (4), IMC 85 (5), JA 10/12 [POU] (6), ICS 60 (7), ICS 95 (8), NAP 25 (9), PA 156 [PER] (10) and TRD 45 (11), and the same locus amplified from four leaves (1–4) from three consecutive flushes of accession IMC 85 (**b**). Sampled leaves are shown in the upper panel and the gel image of the PCR is shown in the lower panel. (**c**) A 193 bp fragment of RT-RNase H region of CaMMV amplified from cocoa accessions GEBP 584/A-F [ADI] (1), PNG 138 (2), SCA 12 (3) and WA 40 [DR] (4). ICS 27 was included as positive control (+Ve) in both assays.

Primer set 1, targeting a 193 bp region of the RT-RNase H region of CaMMV, amplified an expected size band in accessions GEBP 584/A-F [ADI], PNG 138, SCA 12 and WA 40 [DR] (Table 1, Figure 3c). Sequencing of the amplicon amplified from the GEBP 584/A-F [ADI] sample revealed 100% nucleotide identity with ICS 27, ICS 76 and SCA 6 (GenBank accession KX276640) isolates. 

**Figure 4:**
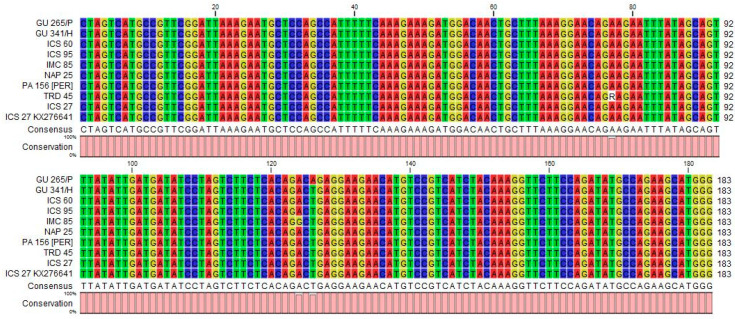
Alignment of 183 bp nucleotide sequences of cacao yellow vein-banding virus (CYVBV) RT-RNase H region obtained in this study from asymptomatic leaf samples from seven cocoa accessions and symptomatic sample of ICS 27 accession received from Trinidad. The CYVBV genome reported in Trinidad (KX276641, ICS 27) is used as reference sequence. The analysis was conducted using CLC sequence viewer (Qiagen, UK).

An expected, the size band of the 183 bp RT-RNase H region of CYVBV was amplified in 11 accessions: GU 144/C, GU 219/F, GU 265/P, GU 341/H, IMC 85, JA 10/12 [POU], ICS 60, ICS 95, NAP 25, PA 156 [PER] and TRD 45 (Table 1, Figure 3a). Alignment of the sequencing data obtained from eight samples revealed that five samples from accessions GU 341/H, ICS 60, ICS 95, NAP 25, PA 156 [PER] were 100% identical with the reference sequence (GenBank accession KX276641). Samples GU 219/F, IMC 85 and TRD 45 varied by one SNP with the reference sequence. The SNP found in TRD 45 is heterozygous in nature (Figure 4).

### 3.4. Effect of Virus Titre and Leaf Age

The brightness of the bands in the CaMMV and CYVBV assays was found to be low, as compared to the positive control; this reflects the low titre of virus present in the sampled leaf. Therefore, PCR was repeated for all positive samples using DNA re-extracted from the same sample, and a sample was considered positive in case of successful amplification in both rounds. Due to the negative correlation between amplification and fragment size, we could not amplify the 861 bp CaMMV fragment in any of the positive accessions, whereas an 803 bp CYVBV fragment was amplified from the IMC 85 sample only.

For the determination of possible variation in virus titre with developmental stages, four leaves from three consecutive flushes were sampled from accession IMC 85. No amplification was found in flush or very old leaf samples. A brighter band was amplified from the fully expanded young leaf, whereas a faint band was found in the old leaf sample (Figure 3b).

### 3.5. LAMP Assay

**Figure 5:**
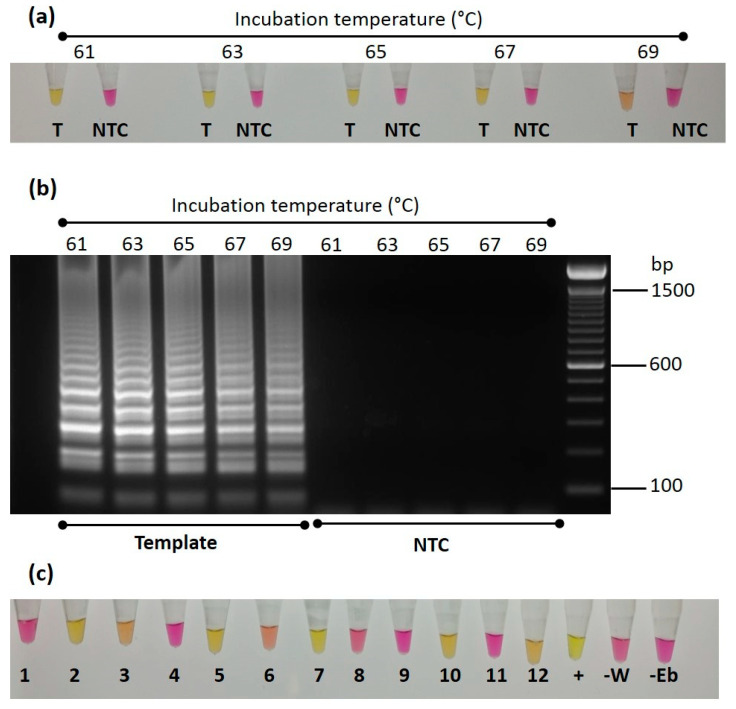
Optimization and testing of colorimetric Loop-mediated isothermal amplification (LAMP) for detection of cacao yellow vein-banding virus (CYVBV). (**a**) LAMP reaction containing 50 ng of DNA of symptomatic ICS 27 sample (T) or without DNA (NTC) were incubated at 61, 63, 65, 67 and 69 °C. After 30 min, samples were removed from the thermal cycler and cooled on ice to stop the reaction. Photograph shows the pink to yellow colour change in the reaction with template DNA. (**b**) The RT-LAMP reaction products were analysed on a 2% agarose gel. The typical band pattern of a successful LAMP reaction was visible in the reactions those contain ICS 27 DNA and showed colour change from pink to yellow. (**c**) Twelve LAMP reactions that contained 50 ng DNA of 12 samples GEBP 509/A-F (1), GU 144/C (2), GU 219/F (3), GU 263/V (4), GU 265/P (5), GU 341/H (6), IMC 85 (7), NA 70 (8), PA 175 [PER] (9), PA 156 [PER] (10), SHRS 09 (11), TRD 45 (12), along with a positive control ICS 27 (+) and two no template controls (-W -Eb), were incubated at 65 °C for 45 min. Photograph shows the pink to yellow colour change in positive samples.

In addition to use of the standard qualitative PCR assay for the detection and identification of Trinidad viruses, a colorimetric LAMP assay was designed for the detection of CYVBV DNA in cocoa accessions. The assay was optimized and validated using the ICS 27 symptomatic sample. For optimization, the LAMP reactions were incubated at 61, 63, 65, 67, 69 °C for 30 min to identify the optimum temperature. High amplification, indicated by a change in the colour of the reaction from pink to yellow, was found at 61, 63, 65 and 67 °C. The change in colour was not very distinct in the reaction incubated at 69 °C; this shows the poor efficiency of the assay to detect CYVBV at elevated temperature. No amplification was found in the no template control reactions at all five temperature conditions (Figure 5a). A 2.5 μL aliquot of the LAMP reaction products was also run on 2% agarose gel for confirmation of amplification. A distinct expected amplicon pattern was found in those samples that showed a colour change from pink to yellow (Figure 5b). For validation of the LAMP assay, a total of 12 samples were tested, including seven samples found positive and five samples found negative for CYVBV in the standard PCR assay. The ICS 27 symptomatic sample was included as a positive control. Two no template control reactions, one with water, and other with elution buffer used during DNA extraction, were also included. The samples were incubated at 65 °C. The positive control reaction turned yellow in 30 min, whereas the sample previously found to be positive in standard PCR took 45 min to change colour. The colours of the reactions of negative samples and no template controls remained unchanged, even after 90 min of incubation (Figure 5c).

### 3.6. Identification of CaMMV and CYVBV Viruses in Genomic Sequencing Datasets of Cocoa

Recent advances in next-generation sequencing (NGS) have led to the unprecedented generation of genomic and transcriptome data that are now available in public data depositories. These data have brought new possibilities to identify coinfecting known and novel virus genomes [25,26]. Therefore, we screened selected genomic datasets from three projects that report sequencing data for cocoa accessions from Brazil, Puerto Rico or Trinidad with the aim of detecting the Trinidad viruses.

#### 3.6.1. Screening of Datasets for CYVBV

**Figure 6:**
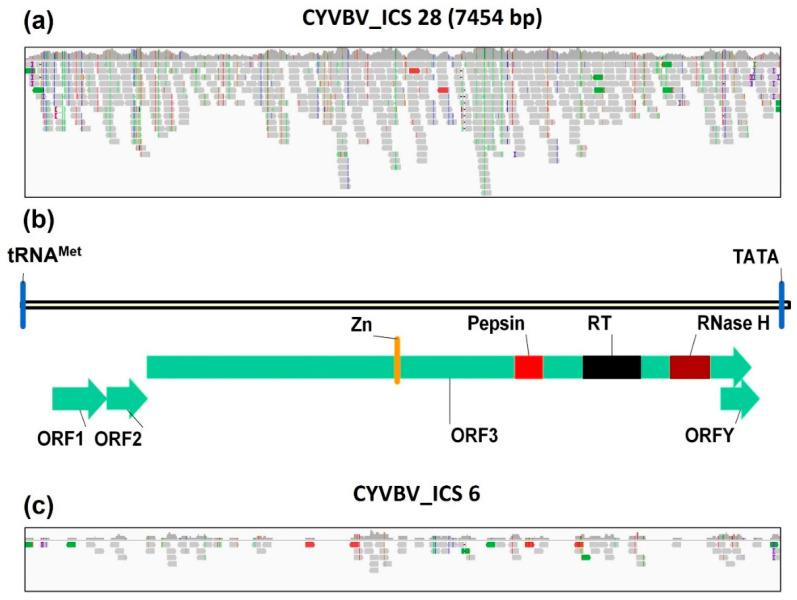
Genome coverage and organization. Sequence reads mapped to reference genome (GenBank Accession KX276641) of cacao yellow vein-banding virus (CYVBV) from SRR7961402 (**a**) and SRR7774312 (**c**) libraries. (**b**) Linear map of CYVBV genome assembled from ICS 28 (SRR7961402) library showing the putative tRNA^met^-binding site (tRNA^met^) and TATA box; predicted open reading frames (ORF)1, ORF2, ORF3 and ORFY; and predicted conserved domains including zinc knuckle finger (Zn), pepsin-like aspartate protease (Pepsin), reverse transcriptase (RT) and ribonuclease H (RNase H).

Mapping of sequence reads against reference genomes identified a complete genome of CYVBV in the SRR7961402 dataset in BioProject PRJNA486150, generated from a leaf sample library of cocoa accession ICS 28 from ICGT, Trinidad. The viral sequence identified had a sequencing coverage of 8.2× (Figure 6a).

A linearized representation of the genome organization is provided in Figure 6b. The identified 7454 bp section of the virus genome shared 98.6% nucleotide identity with the reference genome. The genome consisted of ORFs 1, 2, 3 and Y, comprising 522, 399, 5877 and 378 nt, respectively, and encoding respective putative peptides of 173, 132, 1958 and 125 aa. The genome also contained several hallmark badnavirus conserved motifs including the plant tRNA^Met^ primer binding site and TATA box. A conserved protein domains search using CDD (NCBI) revealed zinc knuckle finger, pepsin-like aspartate protease, reverse transcriptase and ribonuclease H domains in ORF3. Location and organization of these elements predicted in the ICS 28 isolate is consistent with the reference genome.

Screening of datasets in BioProject PRJNA486011 identified only one library containing a CYVBV genome. A considerable number of reads in SRR7774312, a leaf sample library of cocoa accession ICS 6 from ICGT, Trinidad, were mapped to the reference genome, although the coverage was incomplete (Figure 6c). No CYVBV sequences were identified in the 10 datasets in BioProject PRJNA77799.

#### 3.6.2. Screening of Datasets for CaMMV

**Figure 7:**
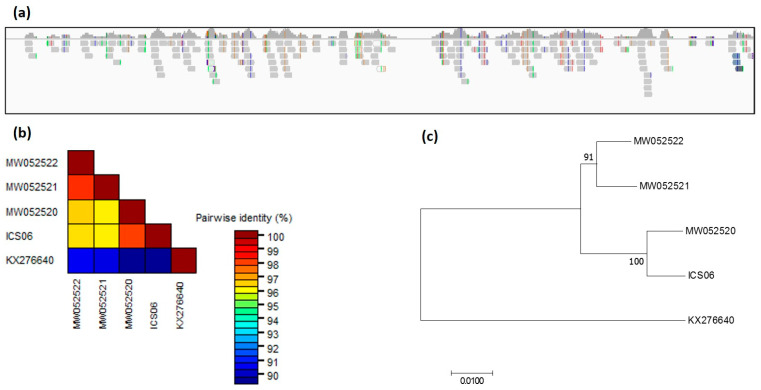
Genome coverage and phylogeny. (**a**) Sequence reads mapped to reference genome (GenBank accession KX276640) of cacao mild mosaic virus (CaMMV) from ICS 6 isolate identified in dataset SRR377712. (**b**) Genetic distances and (**c**) phylogenetic relationship among ICS 6 isolate and CaMMV isolate SCA 06 identified in Trinidad (GenBank accessions KX276640), PR3 isolate discovered in Puerto Rico (GenBank accessions MW052520), and Brazilian isolates BR321 and BR322 (GenBank accessions MW052521, MW052522).

Screening of 10 datasets in BioProject PRJNA77799 identified, in the SRR377712 dataset, a partial genome of CaMMV that covers 74.9% of the CaMMV reference genome (GenBank accession MW052520). This particular library was constructed from a leaf obtained from accession ICS 6 held at the TARS in Puerto Rico (Figure 7a). The consensus sequence was extracted and aligned with four CaMMV genomes. The consensus sequence of the ICS 6 isolate (excluding gaps) showed high nucleotide identity of 98.1% with CaMMV isolate PR 3 from Puerto Rico, as expected. The nucleotide sequence of ICS 6 was 96.3 and 96.5%, identical with CaMMV isolates BR 321 and BR 322 (GenBank accessions MW052521, MW052522) identified in Brazil (Figure 7b,c).

Two hundred WGS datasets in BioProject PRJNA486011 were also screened. A considerable number of reads were aligned to CaMMV reference library in datasets SRR7774196, SRR7774209, SRR7774247 and SRR7774302 generated from accessions SCA 6, EET 395, UF 12 and BR 25, respectively (Table S3). These four accessions studied in this project are held in different cocoa germplasm collections (i.e., BR 25 in Indonesia, EET 395 in ICG-T, SCA 6 in SHRS, Miami and UF 12 in CATIE, Costa Rica). No CaMMV sequences were identified in 30 datasets in BioProject PRJNA486150.

The detection, in all three Bioprojects, of a relatively low number of reads homologous to CYVBV and CaMMV sequences, is not surprising. The datasets in BioProject PRJNA486150 were derived from a reduced representation library sequencing study that was not intended to identify virus sequences but rather was designed for single SNP discovery and allele ancestry mapping in cocoa [15]. Similarly, the other two studies were also not designed to discover virus sequence(s). Therefore, the DNA used to construct the libraries was not enriched for circular DNA with rolling circle amplification (RCA), a common feature of virus identification studies [27]. Similarly, the libraries used in BioProject PRJNA486011 were prepared from DNA that was extracted using a protocol customized for enrichment of high molecular weight DNA, a process that may limit co-extraction of badnavirus DNA.

## 4. Discussion 

Although the badnaviruses that cause Cocoa Swollen Shoot Disease are a major threat to cocoa productivity, much less is known about the related, but genetically distinct CaMMV and CYVBV species, associated with CTV-Strain A and CTV-Strain B. In our study, we identified CaMMV and CYVBV in apparently asymptomatic leaves collected from infected trees of ICS 27 and ICS 76 accessions grown in Trinidad. The CaMMV and CYVBV genomes were initially discovered [3] by using next generation sequencing on samples of ICS 76 and ICS 27 accessions from ICG-T, respectively. In the present study, we made the novel discovery of mixed infection of CaMMV and CYVBV in symptomatic as well as asymptomatic leaf samples from the ICS 27 accession. As the previous study did not mention the identity of the specific ICS 27 tree used, it is quite possible that it might have involved a different tree from that sampled in the present study. The question then arises about the association of symptoms with the detected virus, as the accession contained both viruses but exhibited disease symptoms associated with only one type of virus. In this context, it is appropriate to note the comment of Baker and Dale (1961) [28] that “Trees which have been infected for a long time may be symptomless carriers of the disease, and strain B tends to mask strain A where both occur together”. 

As a complement to the molecular diagnostic approach, the bioinformatic analysis, which comprised a BLAST search in NCBI using an 861 bp sequence of CaMMV as a query, revealed three fragmented, one complete genome and two polyprotein partial cds, identified in the RIM 52 accession in Puerto Rico and one complete genome identified in the VB 1151 accession in Brazil [3,6]. Interestingly, the two polyprotein partial sequences share 89% nucleotide identity with the complete genome isolate identified in the same accession at the same location (Figure 2a). This finding raises the possibility that RIM 52 contains two CaMMV genomes, one of which was not detected in the previous genome discovery study. Endogenous viral elements (EVEs) have been widely reported in plant species [29,30,31,32], including cocoa [2]. It should be noted that neither the standard PCR methods used for CaMMV detection [24], nor the short-read sequencing employed in genome studies [3,6], can differentiate between free virus and EVEs; however, the significantly higher amplification from symptomatic than asymptomatic samples argues against the presence of such integrated elements. A previous relevant study reports hybrid viral/host DNA minicircles of beet curly top Iran virus (BCTIV) in field-grown sugar beet and, by association [33], the presence of such kind of chimeric minicircles in cocoa cannot be ruled out. In addition, it is relevant to note that the occurrence of viruses that do not induce disease symptoms are known in several other crops. For example, the examination of sweet potato germplasm from around the globe has revealed the widespread presence of the badnavirus sweet potato pakakuy virus (SPPV) [34]. This virus, which only occurs at very low titres, is efficiently transmitted through seeds and cuttings and does not induce any obvious symptoms in either sweet potatoes or indicator plants. Similarly, a novel virus has been recently identified in several yam species and tentatively named “yam asymptomatic virus 1” (YaV1) [35]. This virus was first characterized and sequenced from an asymptomatic *Dioscorea alata* plant from Vanuatu. Subsequently, RT-PCR-based screening of a yam germplasm collection conserved in Guadeloupe showed that YaV1 is prevalent in many other *Dioscorea* species but does not cause any symptoms. 

In a broader context, exchange of diverse cocoa germplasm between breeding centres is vital for varietal development through direct introductions and hybridization. Cocoa germplasm has been moved between countries and continents since the 17th century, with the botanic gardens and the quarantine centres playing an increasingly important role in restricting the spread of diseases between cocoa growing regions as awareness of the risks and improved quarantine procedures have developed [1]. Current screening procedures used at ICQC-R use a combination of visual inspection and molecular detection techniques that are effective in detecting infections with CSSV. However, detecting latent or mild virus infections, which produce transient or no visual symptoms in the host plant remains challenging, even with the recent advances in PCR based diagnostic methods due to the inter- and intraspecies diversity of the badnavirus at the genome level. Specifically, the general PCR based approach in cacao virus detection depends upon degenerate primers designed from a highly conserved region, mostly from RT-RNase H region, targeting a region of sufficient length, typically 0.7–1 kb, to facilitate sequencing. Such approaches may lead to false negatives by failed PCR reaction due to the larger fragment size, and false positives by amplifying EVEs by virtue of the degenerate primers used. By using species-specific primers, we successfully identified both CaMMV and CYVBV virus in asymptomatic leaves of accessions held at ICQC-R. The results also demonstrate a negative correlation between amplification and locus size, which necessitates the use of specific primer sets targeting relatively small fragments for efficient amplification in asymptomatic samples with very low virus titre. This is the first report of the identification of CYVBV in asymptomatic samples and the first discovery of CaMMV outside the American continent. 

It should also be noted that our results were not always consistent even across different leaves from the same tree, suggesting low and unequally distributed virus titre in the tree (Figure 3b). We designated an accession positive when we amplified the locus consistently in two independent DNA extractions from the same leaf and, therefore, the number of accessions declared positive represents the minimum number. A range of different approaches have been proposed to overcome the issue of false negatives originating from low copy virus template. One popular technique includes the enrichment of viral DNA with rolling circle amplification prior to detection/identification by conventional PCR or NGS. Recently, a nested PCR protocol has been proposed to identify CaMMV in asymptomatic cocoa samples (https://www.ars.usda.gov/research/publications/publication/?seqNo115=384171). However, despite strict measures being in place, diagnostic laboratories often struggle to avoid false positives results, a problem most commonly caused by the carry-over of DNA from a previous amplification of the same target. Both approaches mentioned above involve two rounds of amplification that can aggravate the problem of false positive results. Certain PCR additives including betaine, DMSO, and PEG can help to enhance amplification [36]. Moreover, the addition of non-specific tail sequences to the 5′-terminus of primers improves the performance of PCR assays [37]. The addition of such a tail can also solve the issue of sequencing of small size amplicons, which are otherwise more efficiently amplified, and reduce the time and the cost of sample preparation for sequencing.

We found sequences of CaMMV and CYVBV in datasets generated from cocoa accessions held in South America, the Caribbean and South East Asia. In a preliminary survey such as this, it is not possible to fully gauge the extent of the virus’ distribution, or the pathways involved in their spread. If we consider the earliest origin of these mild viruses, it is tempting to suggest that this lies in South America, and they are endemic in the centre of diversity of this crop. This suggestion is supported by the fact that badnaviral sequences are known to be integrated into the genome of many cocoa clones [2]; this finding proves that such viruses were present in cocoa prior to, or during, the earliest phase of their domestication. It is plausible that mild or asymptomatic viruses were then transferred to new areas within the Americas, and later outside of the New World as cocoa planting materials were distributed by Europeans keen to establish new plantings to satisfy the growing demand for cocoa products. Although much of the material would have been transported locally in the form of pods or seeds, the recalcitrant nature of cocoa seed means that young plants would have stood a greater chance of surviving long journeys, including the sea voyages, involved in moving material between continents. Indeed, the oldest report of the establishment of cocoa in South East Asia refers to a single plant which survived the journey from Mexico to the Philippines in the mid-seventeenth century (Blanco 1837 cited by Bartley, 2005 [38]). Thus, even if a cacao virus, such as the CSSV, is not seed transmitted, there is still the possibility of spread in whole plants during the earliest movements of cocoa between continents or island groups. Moreover, even where the transfer occurred via a botanic garden in a temperate country, as took place with the material moved from Trinidad to South East Asia in the 1880s, an asymptomatic plant would have remained undetected and there would have been few precautions to prevent contact with potential virus vectors, such as mealybugs. Extensive global exchange and transfer of germplasm has continued until the present day, with an increasing emphasis on the movement of clonal material with known attributes, but it is only in recent years that virus-indexing in a temperate country has been advocated. Thus, it could be speculated that the occurrence of CaMMV in South East Asia reflects an early introduction of infected material from the Americas, followed by spread through the region, and may even be associated with the viral symptoms reported for example in Java from the 1950s. Further global studies in wild and cultivated cocoa would be required to test the validity of this theory.

In addition to the development of PCR-based diagnostics, the present study represents the first report of a LAMP assay for detection of CYVBV in cocoa. This assay successfully detected the virus in an ICS 27 sample, and also differentiated positive samples with very low virus titre from negative samples. However, the time required for the reaction mixture to change colour was longer than the standard 30 min incubation. This finding necessitates the determination of the exact detection limit of the assay. For example, the sensitivity of the assay can be determined by cloning the target amplicon and testing the assay by adding defined number of copies of this cloned artificial template. Moreover, the assay is designed using the two CYVBV sequences generated in this work, and one sequence available in NCBI (GenBank accession KX276641). Overall, the badnaviruses are known for their variation and recombination, and therefore, despite the fact that we targeted the most conserved region to design the assay, variation in this region cannot be ruled out in undiscovered variant(s) of CYVBV. Therefore, the present assay is limited to the detection of the CYVBV Trinidad strain. The sequences of three complete genomes of CaMMV discovered in Puerto Rico and Brazil were made public on 9th of May 2021. As previously mentioned, these sequences vary considerably from the sequence of the isolate reported in Trinidad. Our primers for detection of CaMMV are designed from the Trinidad isolate. We have examined the primer binding site in the newly reported sequence and concluded that these primer sets cannot amplify other variants. In the future it is likely that our LAMP assay could be further improved to improve efficiency and to increase sensitivity, using approaches such as those recently developed in both plant [39] and animal systems [40].

## 5. Conclusions 

In conclusion, this study has identified the presence of two different badnaviruses in a range of asymptomatic cocoa trees. Together with the recent discovery of sequences of related viruses integrated into the genomes of this crop [2], the present findings will allow for the strengthening of existing quarantine procedures and further reduce the risk that viruses, even those which have minimal effect on their host, will evade detection. These issues will be aided by use of the novel LAMP methodology.

## Figures and Tables

**Table 1:** Summary of germplasm in the International Cocoa Quarantine Centre, Reading, United Kingdom that tested positive for cacao mild mosaic virus (CaMMV) or cacao yellow vein-banding virus (CYVBV). A total of 352 accessions were tested.

Sr Number	Name	Accession Number	Donor Genebank
CaMMV			
1	GEBP 584/A-F [ADI]	RUQ 1643	ICG-T
2	PNG 138	RUQ 1354	CIRAD
3	SCA 12	RUQ 1689	ICG-T
4	WA 40 [DR]	RUQ 1283	CIRAD
CYVBV			
1	GU 144/C	RUQ 191	CIRAD
2	GU 219/F	RUQ 768	ICG-T
3	GU 265/P	RUQ 890	ICG-T
4	GU 341/H	RUQ 231	CIRAD
5	IMC 85	RUQ 1666	ICG-T
6	JA 10/12 [POU]	RUQ 456	ICG-T
7	ICS 60	RUQ 959	ICG-T
8	ICS 95	RUQ 1144	CIRAD
9	NAP 25	RUQ 1547	INIAP
10	PA 156 [PER]	RUQ 1531	ICG-T
11	TRD 45	RUQ 1441	ICG-T

CIRAD Centre de Coopération Internationale en Recherche Agronomique pour le Développment, France; ICG-T International Cocoa Genebank, Trinidad, Trinidad and Tobago; INIAP Instituto Nacional de Investigaciones Agropecuarias, Ecuador.

## Data Availability

Sequencing data generated in this study were submitted to NCBI GenBank and have accession numbers MZ419835-MZ419850, Third Party Annotation database accession number BK059205.

## Supplementary Materials

The following are available online at https://www.mdpi.com/article/10.3390/v13112152/s1, Figure S1: Nucleotide alignment of cacao mild mosaic virus (CaMMV) sequences, Figure S2: Nucleotide alignment of cacao yellow vein-banding virus (CYVBV) sequences, Table S1: PCR primers used in detection of cacao mild mosaic virus (CaMMV) and cacao yellow vein-banding virus (CYVBV), Table S2: Loop-Mediated Isothermal Amplification (LAMP) primers used in detection of cacao yellow vein-banding virus; Table S3: Summary of Sequence Read Archive (SRA) experiments analysed in this study.
