# Supplementary material for: Identification of Cacao Mild Mosaic Virus (CaMMV) and Cacao Yellow Vein-Banding Virus (CYVBV) in Cocoa (Theobroma cacao) Germplasm
Source: Viruses. 2021 Oct 26;13(11):2152. doi: 10.3390/v13112152 (PMC8623607; doi:10.3390/v13112152)
Supplement: Supplementary file 1 [file viruses-13-02152-s001.zip › viruses-1363504-supplementary.pdf]

Identification of cacao mild mosaic virus (CaMMV) and cacao yellow vein-banding virus (CYVBV) in cocoa (*Theobroma cacao*) germplasm

Ihsan Ullah, Andrew J. Daymond, Paul Hadley, Michelle J. End, Pathmanathan Umaharan and Jim M. Dunwell

Supplementary figures and tables

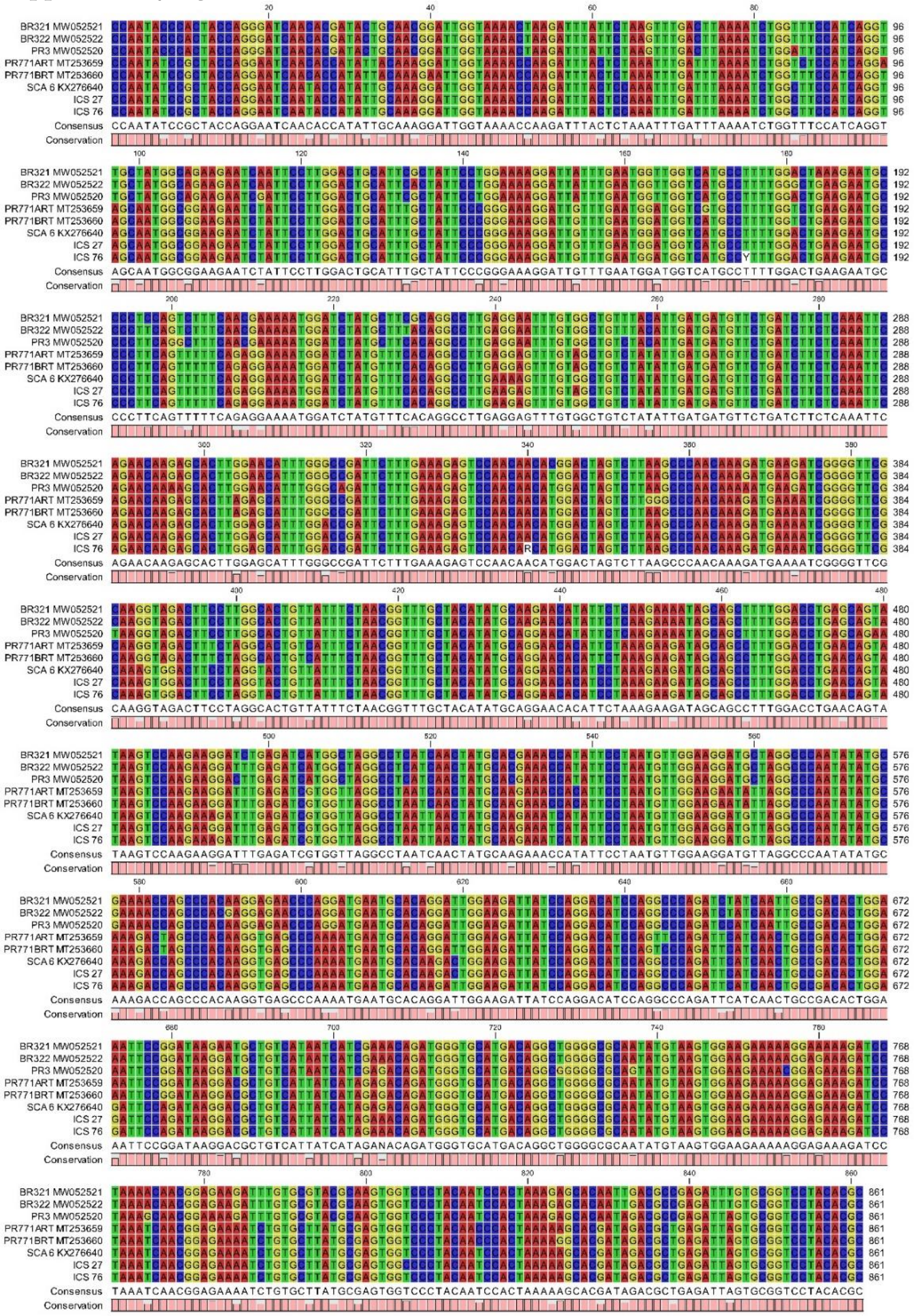

Figure S1. Nucleotide alignment of cacao mild mosaic virus (CaMMV) sequences. Alignment of 861 bp nucleotide sequences CaMMV RT RNase H region obtained in this study (ICS 27; ICS 76); extracted from CaMMV complete genome of isolates from Brazil (BR321 MW052521; BR322 MW052522) Puerto Rico (PR3 MW052520) and Trinidad

(SCA 6 KX276640); and CaMMV polyprotein gene, partial cds of isolates from Puerto Rico (PR771ART MT253659; PR771BRT MT253660). The analysis was conducted using CLC sequence viewer (Qiagen, UK).

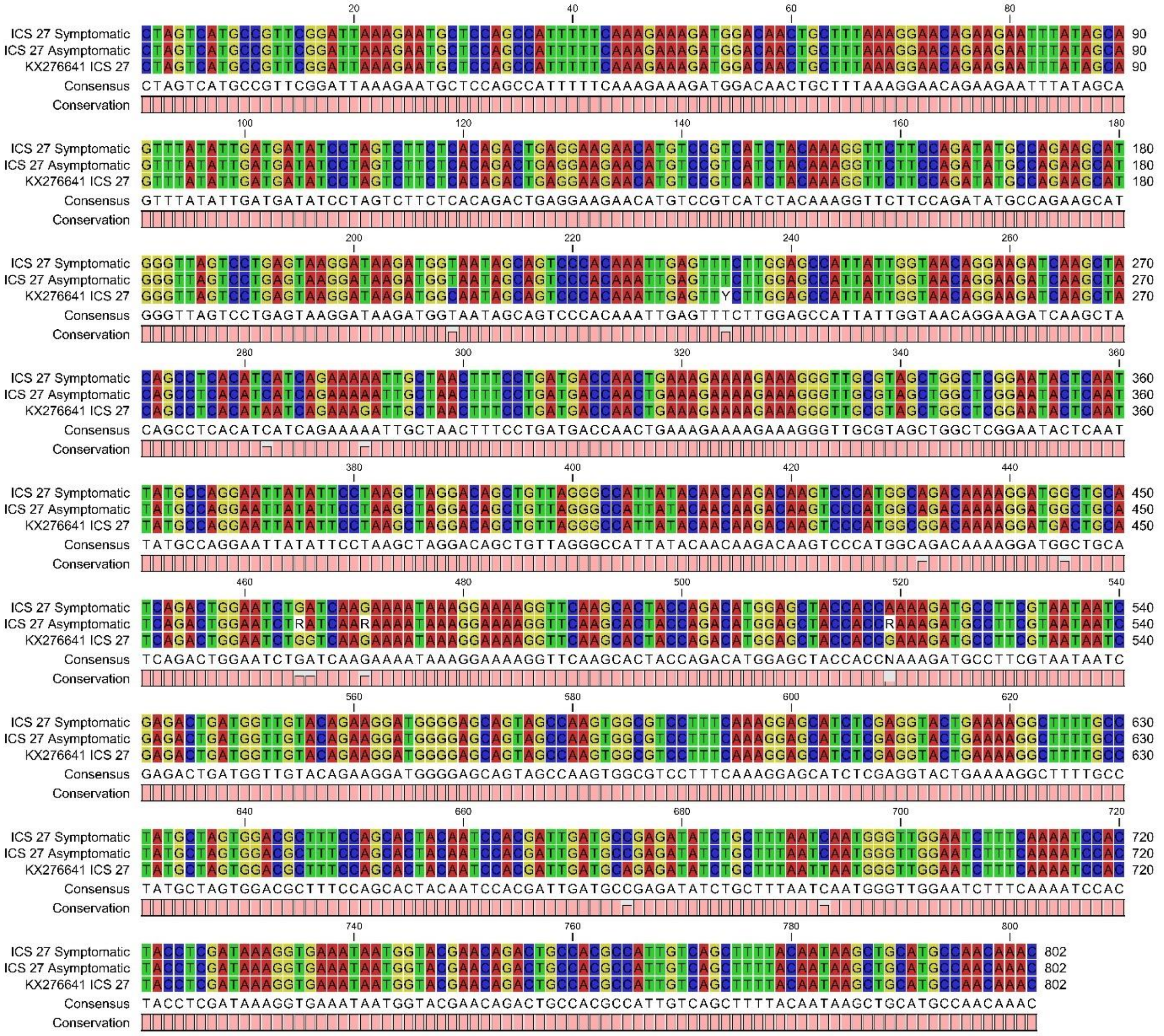

Figure S2. Nucleotide alignment of cacao yellow vein-banding virus (CYVBV) sequences. Alignment of 803 bp nucleotide sequences of CYVBV RT-RNase H region obtained in this study from symptomatic and asymptomatic samples of ICS 27. CYVBV genome reported in Trinidad (KX276641, ICS 27) is used as reference sequence. The analysis was conducted using CLC sequence viewer (Qiagen, UK).

Table S1. PCR primers used in detection of cacao mild mosaic virus (CaMMV) and cacao yellow vein-banding virus (CYVBV).

Specificity	Primer name	Primer sequence	Ta (°C)	Amplicon size (bp)
**CaMMV**	CaMMV2_6292F	TCTGTGCTTATGCGAGTGGT	64	193
	CaMMV2_6484R	CTTAGCCGCGAAGGTTTATG		
	CaMMV1_5507F	CCAATATCCGCTACCAGGAA	62	861
	CaMMV2_6367R	GCGTGTAGGACCGCACTAAT		
**CYVB**V	CYVBV1_5772F	CTAGTCATGCCGTTCGGATT	64	183
	CYVBV1_5954R	CCCATGCTTCTGGCATATCT		
	CYVBV1_5772F	CTAGTCATGCCGTTCGGATT	62	803
	CYVBV2_6573R	GTTTGTTGGCATGCAGCTTA		

Table S2. Loop-Mediated Isothermal Amplification (LAMP) primers used in detection of cacao yellow vein-banding virus (CYBVB).

Primer name	Primer Sequence	Concentration in 10X primermix (μM)
CYVBV_F3	AGACTGGAATCTGATCAAGAA	2
CYVBV_B3	AGCGTCCACTAGCATAGG	2
CYVBV_FIP	AGTCTCGATTATTACGAAGGCATCAAAGGTTCAAGCACTACCAG	16
CYVBV_BIP	TGGTTGTACAGAAGGATGGGGCAAAAGCCTTTTCAGTACCT	16
CYVBV_LF	TTTGGTGGTAGCTCCATGT	4
CYVBV_LB	CAGTAGCCAAGTGGCGTCC	4

Table S3. Summary of Sequence Read Archive (SRA) experiments analysed in this study. Information on Bioproject number, SRA run number, average read length, size of the dataset, name of accession, tree ID (when available) and source of sample material are provided. Red and blue font colour represent the datasets found positive for caco mild mosaic virus (CaMMV) and caco yellow vein banding virus (CYVBV), respectively.

BioProject	Run	Avg. length	Size (Mb)	Sample name	Tree ID	Source of material
PRJNA486150 [1]	SRR7961411	200	17	CL 10/11		ICG-T
	SRR7961410	200	154	CL 10/3		ICG-T
	SRR7961409	200	467	CLM 100		ICG-T
	SRR7961408	200	454	GS 6		ICG-T
	SRR7961407	200	317	GS 61		ICG-T
	SRR7961406	200	168	GU 175/P_1		ICG-T
	SRR7961405	200	34	GU 175/P_2		ICG-T
	SRR7961404	200	101	ICS 1_1		ICG-T
	SRR7961403	200	152	ICS 1_2		ICG-T
	SRR7961402	200	59	ICS 28		ICG-T
	SRR7961427	200	123	ICS 87		ICG-T
	SRR7961426	200	288	IMC 6		ICG-T
	SRR7961429	200	62	IMC 67_1		ICG-T
	SRR7961428	200	12	IMC 67_2		ICG-T
	SRR7961423	200	357	LCT EEN23		ICG-T
	SRR7961422	200	260	LCT EEN246		ICG-T
	SRR7961425	200	272	LCT EEN31		ICG-T
	SRR7961424	200	268	LP 3/29[POU]		ICG-T
	SRR7961431	200	281	LZ 33		ICG-T
	SRR7961430	200	163	M8 [SUR]_1		ICG-T
	SRR7961414	200	479	M8 [SUR]_2		ICG-T
	SRR7961415	200	258	NA 189		ICG-T
	SRR7961416	200	374	NA 26		ICG-T
	SRR7961417	200	206	NA 68		ICG-T
	SRR7961418	200	336	RIM 113[MEX]		ICG-T
	SRR7961419	200	81	SCA 6_1		ICG-T
	SRR7961420	200	260	SCA 6_2		ICG-T
	SRR7961421	200	422	SLC 18		ICG-T
	SRR7961412	200	655	SLC 8		ICG-T
	SRR7961413	200	1667	UF 38		ICG-T
PRJNA77799 [2]	SRR377722	120	2389	*T. grandiflorum*	04-0254	TARS
	SRR377709	120	2145	EET 64	PI 275669	TARS
	SRR376997	120	2317	Amelonado	TARS 16542	TARS
	SRR376998	120	1493	Criollo 22	Criollo-22	SPCL
	SRR377711	120	1545	ICS 1	TARS 16656	TARS
	SRR377712	160	2868	ICS 6	TARS 16658	TARS
	SRR377715	160	2844	ICS 39	TARS 16664	TARS
	SRR377719	160	2588	Pentagonum	TARS 12044	TARS
	SRR377720	118	1607	Scavina 6	MIA 29885	SPCL

	SRR377721	160	2735	Stahel	MIA 27956	TARS
PRJNA486011 [3] *	SRR7774196	172	3996	SCA 6**	Tc16548	USDA-Miami
	SRR7774209	172	8204	EET 395	18186	ICG-T
	SRR7774247	172	8204	UF 12	17434	CATIE
	SRR7774302	119	8819	BR 25		Indonesia
	SRR7774312	172	9197	ICS 6	Tc00551	ICG-T

* All two hundred runs reported in the study were analysed, however, only data for the runs found infected with virus are presented.

**comments from the original study. "Sample with ** is an offset from what SCA 6 should be. It resulted in an admixed individual and researchers interested in looking at SCA 6 should not use this accession as a representative sample from SCA 6 (admixture analysis showed this is a hybrid)".

CATIE	Centro Agronómico Tropical de Investigión y Enseñanza, Costa Rica.
ICG-T	International Cocoa Genebank, Trinidad, Trinidad and Tobago.
SPCL	Sustainable Perennial Crops Laboratory: Beltsville, MD, USA
TARS	Tropical Agriculture Research Station, Puerto Rico.
USDA-Miami	United States Department of Agriculture, Subtropical Horticultural Research Station, Miami, FL, USA.

References

1. Osorio-Guarín, J.A.; Quackenbush, C.R.; Cornejo, O.E. Ancestry informative alleles captured with reduced representation library sequencing in *Theobroma cacao*. *PloS one* **2018**, *13*, *10*. doi: 10.1371/journal.pone.0203973
2. Kane, N.; Sveinsson, S.; Dempewolf, H.; Yang, J.Y.; Zhang, D.; Engels, J.M.; Cronk, Q. Ultra-barcoding in cacao (Theobroma spp.; Malvaceae) using whole chloroplast genomes and nuclear ribosomal DNA. *Am J Bot*. **2012,** *99*, 2. doi: 10.3732/ajb.1100570.
3. Cornejo, O.E.; Yee, M.C.; Dominguez, V.; Andrews, M.; Sockell, A.; Strandberg, E.; Livingstone, D.3rd; Stack, C.; Romero, A.; Umaharan, P.; Royaert, S.; Tawari, N.R.; Ng, P.; Gutierrez, O.; Phillips, W.; Mockaitis, K.; Bustamante, C.D.; Motamayor, J.C. Population genomic analyses of the chocolate tree, *Theobroma cacao* L., provide insights into its domestication process. *Commun biol.* **2018,** *1*, 167. doi: 10.1038/s42003-018-0168-6
